# Fracture toughness of schist, amphibolite, and rhyolite from the Sanford Underground Research Facility (SURF), Lead, South Dakota

**DOI:** 10.1038/s41598-022-20031-y

**Published:** 2022-09-24

**Authors:** Ben Jahnke, Casey Ruplinger, Charlotte E. Bate, Maciej Trzeciak, Hiroki Sone, Herbert F. Wang

**Affiliations:** 1 University of Wisconsin-Madison, Madison, WI USA; 2 Texas A&M University, College Station, TX USA

**Keywords:** Solid Earth sciences, Energy science and technology, Engineering

## Abstract

The Cracked Chevron Notched Brazilian Disc (CCNBD) method was selected for Mode I fracture toughness tests on Poorman schist, Yates amphibolite, and rhyolite dikes from the EGS Collab site at the SURF in Lead, South Dakota. The effects of lithology, anisotropy, and loading rate were investigated. Fracture toughness was greatest in amphibolite, with schist and rhyolite having similar toughness values (${K}_{amphibolite}$ > ${K}_{rhyolite}$ ≈ ${K}_{schist}$). The effects of anisotropy on fracture toughness were investigated in the foliated schist samples. Schist samples were prepared in three geometries (divider, arrester, and short transverse) which controlled how the fracture would propagate relative to foliations. The divider geometry was strongest and short transverse geometry was the weakest (${K}_{divider}$ > ${K}_{arrester}$ > ${K}_{short transverse}$). Fracture toughness was observed to decrease with decreasing loading rate. Optical and SEM microscopy revealed that for the short transverse geometry, fractures tended to propagate along grain boundaries, whereas in arrester and divider geometries fractures tended to propagate through grains. In foliated samples, the tortuosity of the fracture observed in thin section was greater in arrester and divider geometries than in short transverse geometries.

## Introduction

**Figure 1:**
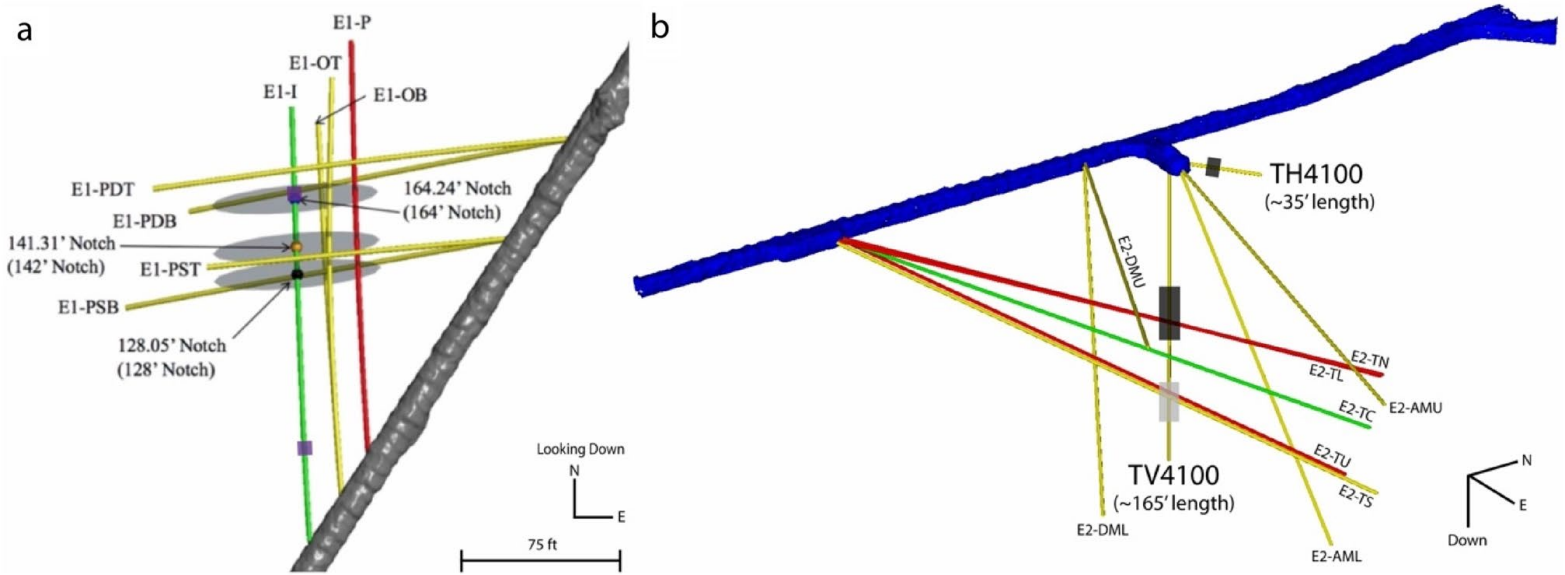
Borehole geometry of testbeds at the SURF. Borehole color indicates utility (green = injection, red = production, yellow = observation). (**a**) 4850-foot level (figure modified from White et al. 2019)^2^. Purple shaded regions indicate locations of schist samples. (**b**) 4100-foot level (figure modified from Kneafsey et al. 2021)^3^. Black shaded regions indicate locations of amphibolite samples; gray shaded regions indicate locations of rhyolite samples.

The EGS Collaboration (EGS Collab) project had the goal to better understand fracture stimulation methods, fracture geometries, and processes controlling heat transfer^1^ in enhanced geothermal systems. Toward this goal, two test beds with boreholes spaced 10–20 m apart, and 70–80 m in length were drilled and instrumented at depths of over one kilometer (Fig. 1). Experiment 1 focused on hydraulically fracturing an injection borehole and connecting it to a production borehole. Fracture toughness is an important mechanical property that quantifies the rock formation’s resistance to Mode I crack propagation.

**Figure 2:**
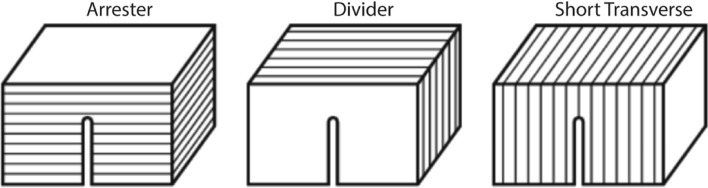
Principal notch orientations with respect to foliation planes. Figure reproduced from Chong et al. 1987^12^.

The work presented in this paper investigates the effects of lithology, anisotropy, and loading rate on the Mode I fracture toughness of the formations at the EGS Collab sites. Experiment 1’s testbed is in the Poorman schist (Fig. 1a), which has foliations that can be observed in planar and tightly folded orientations. These folds occur in a range of length scale from centimeters to meters^4^. Experiment 2’s testbed is in the Yates amphibolite, which contains rhyolite dikes (Fig. 1b). The rhyolite and amphibolite are seemingly homogenous units, with small calcite veinlets present in the amphibolite. Previous studies have shown that fracture propagation is influenced by anisotropy^5^. Furthermore, transversely isotropic rocks have shown that the fracture toughness depends on the relative orientations of mineral alignment and microdefects relative to the direction of fracture propagation^4,6–9^. Tests using foliated schist from the Experiment 1 site were compared with the homogenous-appearing amphibolite and rhyolite from the Experiment 2 site. The Cracked Chevron Notched Brazilian Disc (CCNBD) geometry was selected to measure Mode I fracture toughness, following the International Society of Rock Mechanics (ISRM) suggested method^10^. Since the CCNBD method allows for notch orientation to be adjusted with respect to rock texture^11^, fracture toughness can be determined with respect to anisotropy. CCNBD samples also offer simplified sample preparation and testing setups, high failure loads, and a range of valid sample dimensions. We prepared CCNBD samples in three orientations: arrester, divider, and short transverse (Fig. 2). The three orientations can be described by the notch orientation relative to foliation planes (Supplementary Table S1). This study incorporates visual observations of fracture geometries at the microscale using optical and electron microscopy to better understand the mechanisms resulting in fracture toughness anisotropy.

## Sample selection and preparation

Drilled cores were removed from boreholes at the sites of Experiment 1 and 2. At the 4850-foot depth level, schist cores were recovered from the sub-horizontal borehole, E1-I, at depths of 39′–44′ and 154′–159′ into the borehole. At the 4100-foot depth level, both rhyolite and amphibolite cores were recovered. Rhyolite cores were recovered from the vertical borehole, TV4100, at depths of 120′–135′ into the borehole. Amphibolite cores were recovered from both sub-horizontal and vertical boreholes, TH4100 and TV4100, respectively. Amphibolite cores from TH4100 were recovered at depths of 7′–12′ into the borehole, and cores from TV4100 were recovered at depths of 65′–95′ into the borehole.

**Figure 3:**
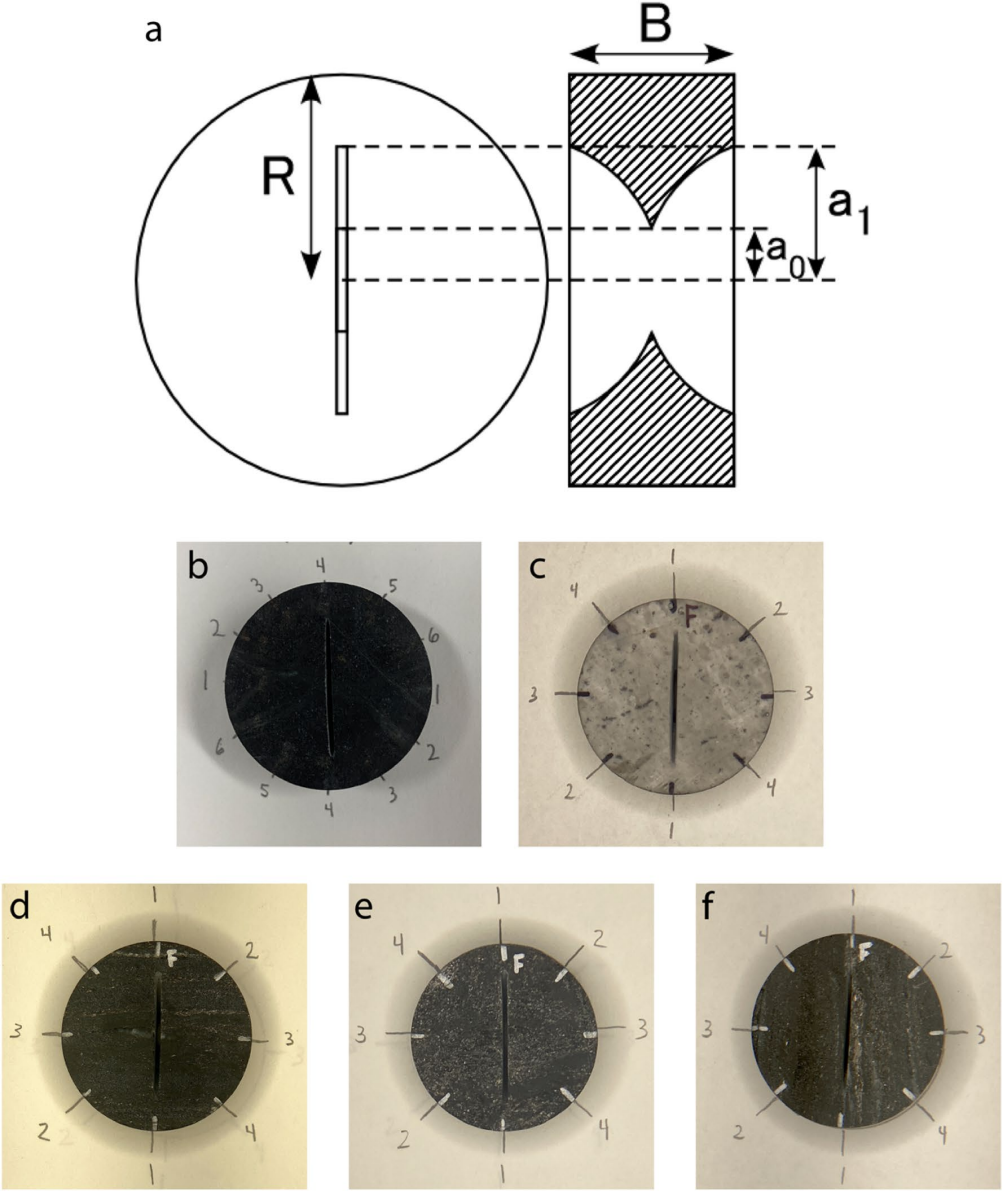
(**a**) CCNBD sample geometry. a_0_ is half the initial chevron notch length, a_1_ is half the final chevron notch length, B is the disc thickness, and R is the disc radius. Figure modified from Fowell et al.^10^. (**b**–**f**) Photos of representative samples from each sample group: (**b**) amphibolite, (**c**) rhyolite, (**d**) schist–arrester, (**e**) schist–divider, (**f**) schist–short transverse. The numbers surrounding the amphibolite disc indicate where travel time measurements were made. The numbers surrounding the rhyolite and schist discs indicate where diametrical measurements were made.

Sub-cores of 1.5-in. (38.1 mm) diameter were removed from the 2.4-in. (61.0 mm) diameter drilled cores. As there was no fabric from visual inspection of the amphibolite and rhyolite cores, sub-cores were removed without any specific orientation. Schist sub-cores were removed either parallel or perpendicular to foliation. The sub-cores were then sliced into 0.5-in. (12.7 mm) thick discs. A custom-made device was used to cut chevron notches into each face of the discs following an established method^13^ (Fig. 3a). The device used a 1.2-in. (30.5 mm) diameter diamond blade attached to a rotary tool. The discs were fastened into a holder which was lowered onto the blade, guided by three vertical rods, with the disc faces perpendicular to the blade. The depth of the cut was controlled by a pair of nuts tightened on a vertically oriented, threaded rod attached to the base of the device. The nuts limited the depth to which the holder could be lowered. The notch lengths were determined using the range of valid geometric dimensions established by the ISRM^10^.

Notch orientations were determined by the visual indicators of anisotropy observed in each of the discs. Notches were cut into one of three orientations in the schist discs depending on the orientation of foliation planes with respect to the disc faces. If foliation planes were perpendicular to the disc faces, notches were cut either perpendicular or parallel to foliation planes to create arrester and short transverse geometries, respectively. If foliation planes were parallel to the disc faces, notches were cut in any direction to create divider geometries. Notch orientations were randomly cut into the rhyolite and some amphibolite discs due to lack of visual anisotropy. However, for six amphibolite samples, notches were cut in either the direction of fastest or slowest P-wave velocity, where P-wave velocities were measured every 30° around their circumference. In total, five sample groups were created for CCNBD testing from the amphibolite, rhyolite, and schist sub-cores (Fig. 3b–f).

It is known that the orientation of Mode I fractures at the macroscopic scale is controlled by stress orientation^14^. However, we also wanted to investigate how the fracture paths and surfaces might be influenced by internal structure at the grain scale. As fractures are assumed to initiate at the notch tips in the middle of the CCNBDs, thin sections were prepared to exhibit the plane containing both tips to observe the nature of fracture initiation. After completion of fracture toughness tests, thin sections were prepared for at least one sample from each of the five sample groups. Thin sections were prepared parallel to the faces of the discs, as close as possible to the plane containing both notch tips. Fracture geometry, fabric, and mineralogy were examined using both optical and scanning electron microscopy.

## Laboratory methods

To check for anisotropy in the amphibolite samples before cutting notches, we measured P-wave velocities across the disc diameters using acoustic transducers (Supplementary Fig. S1a). Six diametrical travel time measurements were made per disc, with each measurement separated by 30° around their circumference. We saw a sinusoidal velocity profile for each of the six discs, with the minimum and maximum P-wave velocity separated by 90°. Three pairs of amphibolite discs had similar velocity profiles.

A servo-controlled triaxial testing system was used to conduct the fracture toughness tests at room temperature without confining pressure. Custom jaws made for Brazilian discs following ISRM specifications were used to apply a diametrical compressional load to the test samples (Supplementary Fig. S1b). Vertical displacement of the upper jaw relative to the lower jaw was recorded by two linear variable differential transducers (LVDT), while horizontal expansion was recorded by a displacement transducer based on a strain gage.

**Figure 4:**
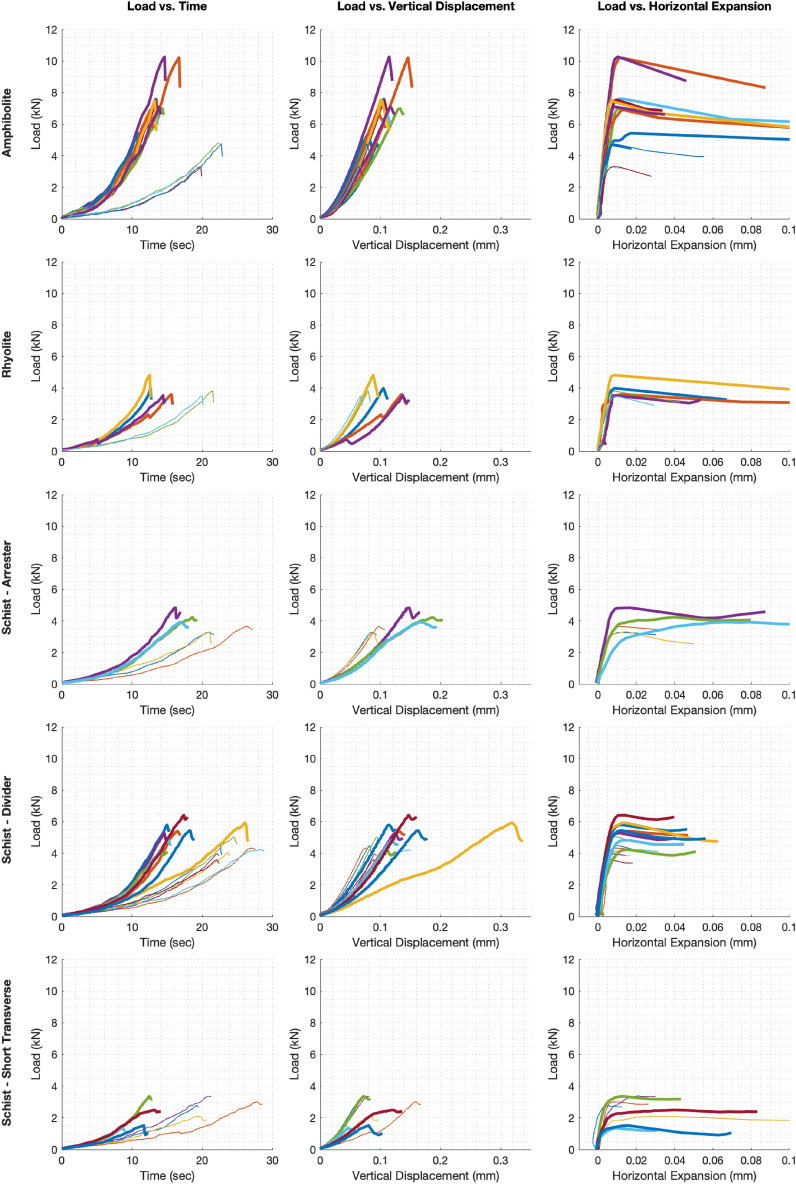
Results from fracture toughness loading tests. Rows, from top to bottom: amphibolite, rhyolite, schist–arrester, schist–divider, schist–short transverse. Bold and thin curves indicate samples loaded at rates of 20 μm/s and 10 μm/s, respectively.

Tests followed a three-phase sequence. In the first phase, the load cell advanced downward until the load reached 0.1 kN. This ensured that the load cell was in full contact with the sample assembly prior to the start of the second phase. In the second phase, the sample was loaded under piston displacement control, at a rate of either 10 or 20 μm/s. Structural failure was determined by the load vs. horizontal displacement graph displayed on the testing software. A load drop associated with an abrupt expansion of the horizontal displacement transducer, denoted as horizontal expansion, was used as indication that a fracture had formed (Supplementary Fig. S1c). Throughout the entirety of the test, the load, vertical displacement, and horizontal displacement were recorded at a sampling rate of 10 Hz (Fig. 4).

## Results

### Loading behavior

Samples were observed to be more compliant at the onset of loading due to microcracks within the rock, resulting in the load increasing relatively slowly. Once microcracks closed, the load increased more rapidly until structural failure. Before an abrupt load drop was observed, there were only minimal amounts of horizontal expansion. The abrupt load drop was associated with significant horizontal expansion. We used this as the indication of structural failure and fracture propagation. Structural failure of the amphibolite occurred at horizontal expansions between 0.01 and 0.05% strain, rhyolite between 0.02 and 0.04% strain, schist–arrester between 0.03 and 0.18% strain, schist–divider between 0.02 and 0.05% strain, and schist–short transverse between 0.01 and 0.10% strain. Vertical deformation scaled nearly linearly with respect to load from test initiation to the maximum load, followed by unstable crack propagation until a local load minimum was reached, consistent with expected behavior^15^. Because the testing procedure limited propagation to a single fracture, no further fracture formations were observed. Variation in the stiffness within the schist groups may be due to slight differences in the foliation orientation^16^. Structural failure of amphibolite occurred between vertical strains of 0.17–0.38%, rhyolite between 0.18 and 0.36%, schist–arrester between 0.22 and 0.49%, schist–divider between 0.19 and 0.84%, and schist–short transverse between 0.12 and 0.41%.

### Fracture toughness results

Mode I fracture toughness of CCNBD samples was calculated from the ISRM suggested method^17^:

1 $${K}_{IC}=\frac{{P}_{max}}{B\sqrt{R}}{Y}_{min}^{*}$$

where ${K}_{IC}$ is fracture toughness, ${P}_{max}$ is the maximum diametrical load, $B$ is the sample thickness, $R$ is the sample radius, and ${Y}_{min}^{*}$ is the critical (minimum) dimensionless stress intensity factor, which is dependent on the sample geometry and is calculated using the following formula:

2 $${Y}_{min}^{*}=u{e}^{v{\alpha }_{1}}$$

where $u$ and $v$ are geometric constants derived from α_0_, α_1_, and α_B_, which are defined as:

3 $${\alpha }_{0}=\frac{{a}_{0}}{R}$$

4 $${\alpha }_{1}=\frac{{a}_{1}}{R}$$

5 $${\alpha }_{B}=\frac{B}{R}$$

where ${a}_{0}$ is half the initial chevron notch length and ${a}_{1}$ is half the final chevron notch length (Fig. 3a). Using the ISRM suggested method^10^, $u$ and $v$ were found through linear interpolation using the geometric sample dimensions.

**Figure 5:**
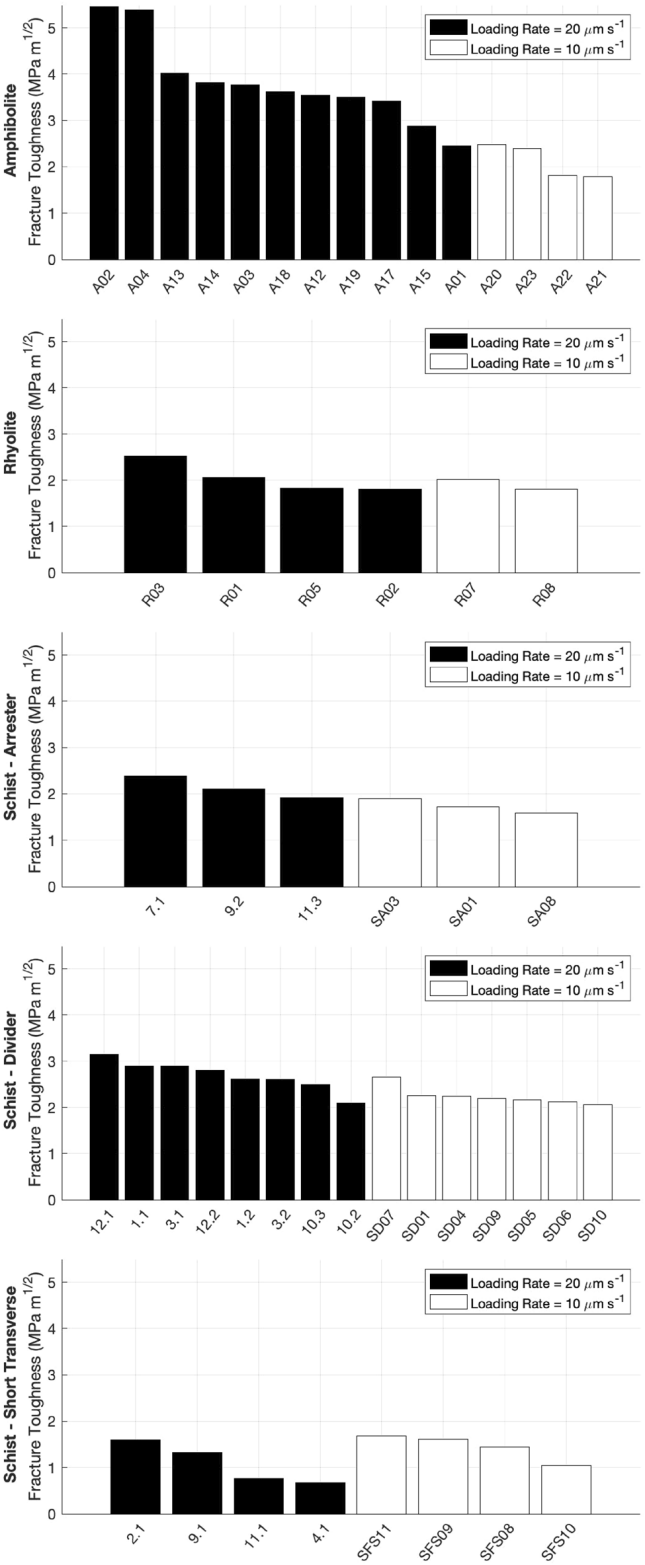
Fracture toughness values from all tests. Black bars indicate a loading rate of 20 μm/s, white bars indicate a loading rate of 10 μm/s.

Using Eqs. (2)–(5), ${Y}_{min}^{*}$ was calculated for each sample based on geometric measurements taken prior to each fracture toughness test. After calculating ${Y}_{min}^{*}$, the fracture toughness was calculated using Eq. (1) (Fig. 5) (Supplementary Table S2). In some cases, relatively significant horizontal expansion occurred prior to a load drop. This is accounted for by ${Y}_{min}^{*}$^18^, which assumes stable propagation prior to the load drop after which the fracture propagates unstably.

It has been shown that stress intensity factors are influenced by material anisotropy which can influence the calculation of fracture toughness^19^. However, we used the solutions developed for isotropic materials^17^ because stress intensity factors for CCNBD geometries in anisotropic materials have not been developed yet.

Of the three lithologies, fracture toughness was greatest in the amphibolite, with the rhyolite and schist having comparable toughness values (Supplementary Table S3). For a loading rate of 20 μm/s, the mean toughnesses (in MPa m^1/2^) of the amphibolite and rhyolite samples were 3.80 ± 0.91 and 2.06 ± 0.34, respectively. For a loading rate of 10 μm/s, the mean toughnesses (in MPa m^1/2^) of the amphibolite and rhyolite samples were 2.12 ± 0.37 and 1.91 ± 0.15, respectively.

The fracture toughness of the schist samples was greatest in the divider, and weakest in short transverse geometry. For a loading rate of 20 μm/s, the mean toughnesses (in MPa m^1/2^) of the arrester, divider, and short transverse geometries were 2.14 ± 0.23, 2.70 ± 0.32, and 1.09 ± 0.44, respectively. For a loading rate of 10 μm/s, the mean toughness (in MPa m^1/2^) of the arrester, divider, and short transverse geometries were 1.73 ± 0.15, 2.24 ± 0.20, and 1.44 ± 0.28, respectively.

### Microstructural observations

#### Optical microscope

**Figure 6:**
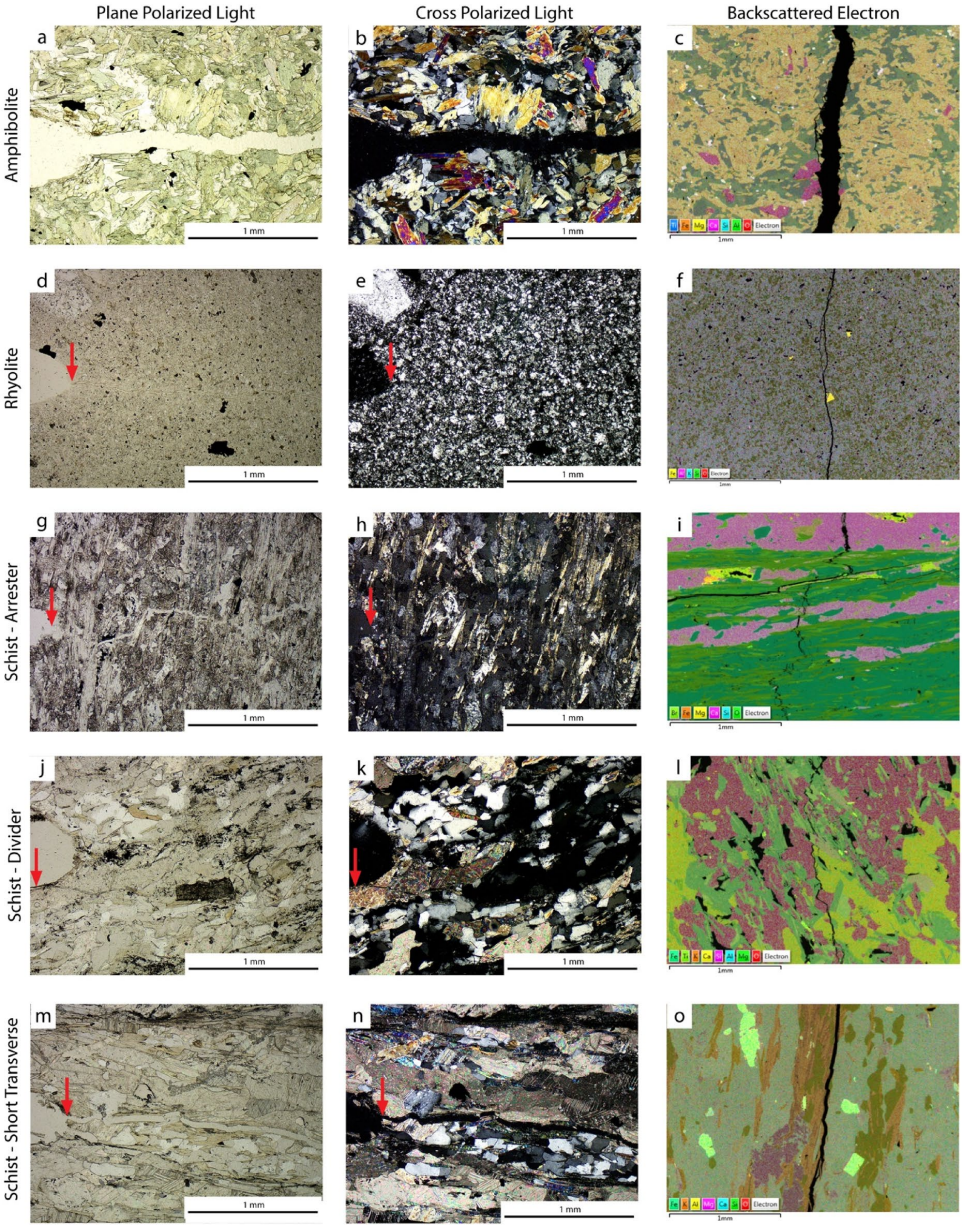
Thin section images, from an optical microscope under plane-polarized and cross-polarized light, and a BSE-EDS stacked image from a scanning electron microscope for each sample group. Arrows indicate the location of fracture initiation.

Fractures were observed using an optical microscope under plane-polarized and cross-polarized light (Fig. 6). These observations provided insights on microstructure of the rock and fracture.

- Amphibolite samples (Fig. 6a,b) showed randomly oriented needle-shaped grains with no apparent fabric. Fractures initiated at the center of the notch tip and were relatively straight and intragranular.
- Rhyolite samples (Fig. 6d,e) showed a fine-grained microcrystalline texture with no visual anisotropy. Fractures initiated at the center of the notch tip and were relatively straight and intragranular.
- Schist–arrester samples (Fig. 6g,h) showed foliation perpendicular to the fracture. Fractures initiated at the center of the notch tip, were tortuous and primarily intragranular, but sometimes intergranular and perpendicular to the overall fracture propagation directions.
- Schist–divider samples (Fig. 6j,k) showed randomly oriented grains because the foliation planes are parallel to the thin section plane. Fractures initiated slightly off-center from the notch tip, and the tortuosity of the fracture is difficult to observe once the fracture propagated from the notch tip. The fracture might be intergranular; however, it is difficult to distinguish in the thin section.
- Schist–short transverse samples (Fig. 6m,n) showed foliation parallel to the fracture. Fractures initiated at the center of the notch tip and were relatively straight and intergranular.

#### Scanning electron microscope

Fractures were investigated using a scanning electron microscope. Energy dispersive X-ray spectroscopy (EDS) mapping provided confirmation of the mineralogical compositions of the formations^20^ (Fig. 6).

- The Yates amphibolite contained hornblende and plagioclase with small amounts of calcite and ilmenite (Fig. 6c).
- The rhyolite showed an aphanitic texture and primary minerals included fine-grained quartz and feldspar, with sparse mafic phenocrysts (Fig. 6f).
- The Poorman schist contained sericite, biotite, calcite, muscovite, pyrite, and dolomite (Fig. 6i,l,o).

**Figure 7:**
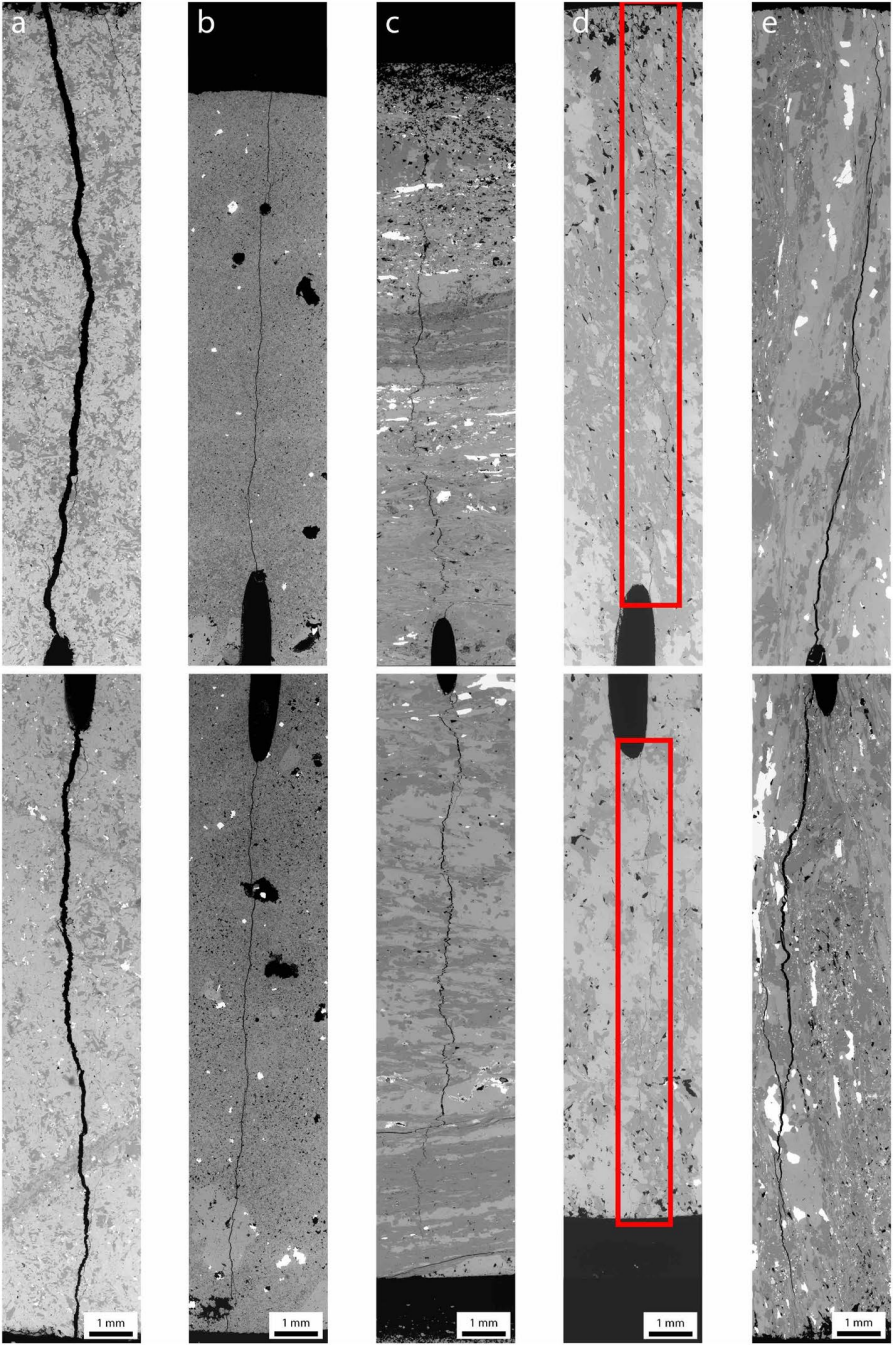
BSE images displaying the entire length of the fracture for each of the five sample groups: (**a**) amphibolite (A14), (**b**) rhyolite (R08), (**c**) schist–arrester (SA08), (**d**) schist–divider (SD04), (**e**) schist–short transverse (SFS10). Outline indicates region of fracture.

Backscattered electron (BSE) imaging was done to observe the entire fracture length and geometry in greater detail (Fig. 7).

- Amphibolite samples (Fig. 7a) showed relatively straight and smooth fracture paths initiating from the center of both notch tips. Fracture propagation appeared to be primarily intragranular. Apparent fracture aperture in these samples was the widest amongst all sample groups.
- Rhyolite samples (Fig. 7b) showed relatively straight and smooth fracture paths initiating from the center of both notch tips. Fracture propagation appeared to be primarily intragranular. Apparent fracture aperture was relatively thin.
- Schist–arrester samples (Fig. 7c) showed relatively tortuous fracture paths initiating from the center of both notch tips. The overall trend of the fracture was to propagate perpendicular to foliation, although locally the fracture follows grain boundaries, thus increasing tortuosity. Secondary fractures parallel to foliation propagated a considerable length from the primary fracture. Apparent fracture aperture was relatively thin.
- Schist–divider samples (Fig. 7d) showed relatively tortuous fracture paths initiating slightly offset from the center of notch tips. Fracture propagation appeared to be primarily intragranular with lesser amounts of intergranular fracturing. Apparent fracture aperture was the thinnest amongst all sample groups.
- Schist–short transverse samples (Fig. 7e) showed relatively straight and smooth fracture paths initiating both at the center and slightly offset from the center of notch tips. Fracture propagations appeared to be primarily intergranular. The fracture was relatively straight and smooth as fracture propagation is primarily intergranular. Apparent fracture aperture was relatively wide; widest amongst the three specified fracture orientations.

## Discussion

### Effects of lithology, anisotropy, and loading rate on fracture toughness

Fracture toughness appeared to be influenced by lithology. The schist samples were primarily composed of phyllosilicates; thus, planes of weakness were present for preferential fracture propagation. As the amphibolite samples were composed primarily of randomly oriented hornblende and plagioclase crystals, there were no preferential planes of weakness for the fractures to propagate through. This forced the fractures to propagate through the hornblende and plagioclase, resulting in a relatively high fracture toughness and relatively straight fracture paths. Rhyolite was also made up of crystalline material but at a much finer scale than the amphibolite. Grain size appeared to influence fracture toughness, as a decrease in grain size makes it easier for fractures to follow the highest stress concentrations since there are more grain boundaries. Therefore, small grain size is likely the reason for the relatively straight fracture paths observed in the rhyolites.

Fracture toughness appeared to be influenced by foliation and fabric revealed by velocity anisotropy. The magnitude of fracture toughness for the three geometries of schist CCNBDs is likely related to the fracture propagation resistance. The foliated nature of the schist provided planes of weakness for preferential fracture propagation. In the short transverse geometry, fractures propagated along planes parallel to foliation between phyllosilicate minerals (Figs. 6o, 7e). These foliation planes have been found to be relatively weak planes in the Poorman schist^16^. A lower fracture tortuosity was also observed in the short transverse samples when compared to the arrester samples, consistent with previous findings^21^. More energy is required for intragranular fracturing than intergranular fracturing due to internal mineral strength, resulting in greater fracture toughness. In the arrester orientation, fractures propagated through foliation planes sequentially, whereas in the divider orientation, fractures propagated through all foliation planes simultaneously. It is easier to sequentially propagate fractures through individual planes in the arrester geometry, as the foliations in the divider geometry act as a composite layer consisting of both relatively weak and strong planes.

**Figure 8:**
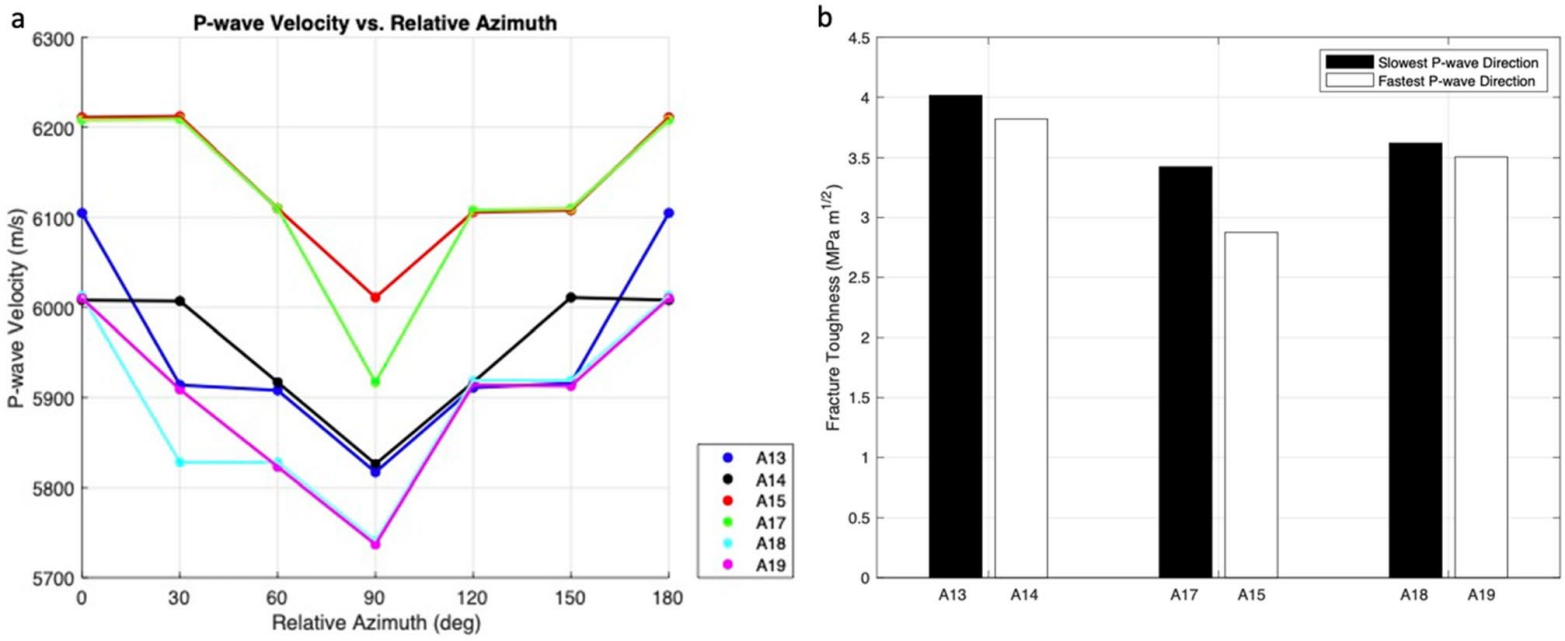
(**a**) P-wave velocity as a function of direction of six amphibolite discs without notches. Relative azimuth is based on fastest direction. (**b**) Fracture toughness values for pairs of amphibolite discs whose velocity variations with notch direction are similar.

Although no visible fabric was apparent in the amphibolite samples, there was a correlation between fracture toughness and notch orientation with respect to the velocity anisotropy (Fig. 8). Within each of the three comparison pairs, fracture toughness appeared to vary relative to anisotropy, as observed in other studies^22^. However, the natural sample variability appeared to have a greater control on fracture toughness than the velocity anisotropy. P-wave anisotropy could be due to microstructure such as preferred mineral orientation or alignment of microcracks. Thus, the fastest direction of P-wave propagation is parallel to planes defining anisotropy, and the slowest direction of P-wave propagation is sub-perpendicular to the planes defining anisotropy. Therefore, notches oriented in the fastest P-wave direction can be thought of as mixed short transverse-divider, whereas notches oriented in the slowest P-wave direction can be thought of as mixed arrester-divider. The subtle velocity anisotropy in the amphibolite samples shows a smaller relative difference in fracture toughness between mixed short transverse-divider and mixed arrester-divider geometries (3–25%) than fracture toughness between short transverse and arrester/divider geometries for schist (75–200%). The effects of anisotropy on fracture toughness observed for schist and amphibolite suggest that fracture toughness is lower when fractures propagate parallel to transversely isotropic planes. These observed trends are consistent with previous studies on anisotropic shales^6–9^ (Supplementary Table S4).

Fracture toughness was observed to decrease with decreasing loading rate. When the loading rate was halved, in our case from 20 to 10 μm/s, there was a systematic decrease in the fracture toughness for all our sample groups except for short transverse samples. The decrease with slower loading rate is expected because fractures have additional time to propagate stably and weaken the rock before unstable propagation^23^. Possible causes for the larger fracture toughness for short transverse samples tested at a loading rate of 10 μm/s include stronger foliation planes, lower foliation aspect ratios, and/or foliation planes not being perfectly planar and/or perpendicular to disc faces.

### Implications for the field-scale

Enhanced geothermal systems use hydraulic stimulation to increase the permeability of hot, low permeability rocks. The hydraulic pressure required to stimulate low permeability rocks is a function of the difference between the hydraulic pressure and magnitude of the least principal stress^24^. Therefore, pressures required to initiate hydraulic fractures will directly scale with fracture toughness. The amphibolite samples were observed to have the greatest fracture toughness; therefore, hydraulic stimulation in amphibolite will require greater hydraulic pressures than in the rhyolite and schist.

The divider samples were observed to have the greatest fracture toughness, and, therefore, hydraulic stimulation will require greater hydraulic pressures than in the arrester and short transverse geometries.

Our results show that the tortuosity and roughness of fractures depends on whether the fracture propagates parallel or perpendicular to foliation planes. In the arrester geometry, fractures were observed to propagate primarily perpendicular to foliation, locally parallel to foliation when possible, and even instances where propagation was diverted to be parallel to foliation (Fig. 7c). Visual observations suggest that fractures that propagated in the short transverse and divider geometries were not as tortuous as fractures that propagated in the arrester geometry. The variety of possible fracture propagation orientations in the arrester geometry may increase the tortuosity of the fracture, and thus increase the surface area of the rock matrix forming the fracture surfaces. Therefore, fractures propagating perpendicular to foliation planes could possibly result in the greatest increase in surface area per unit length. As a result of the increased tortuosity and roughness, heat exchange between circulating fluid and host rock should have a higher efficiency^25^.

The tortuous Mode I fractures of an arrester geometry are comparable to the corrugated tensile fractures in a 3D-printed “geo-architected rock”^26^. The corrugated surface is a consequence of mineral lineation parallel to layering. Depending on fluid flow direction relative to the strike of the corrugated surface, permeability may be anisotropic within the fracture plane. Flow is obstructed less when flowing parallel, rather than perpendicular, to the strike of corrugated surfaces. If flow is perpendicular to the strike, the permeability will be relatively low as viscous dissipation will be enhanced due to frictional losses and increased length of flow path.

## Conclusion

We performed Mode I fracture toughness tests using Cracked Chevron Notched Brazilian Disc (CCNBD) samples of amphibolite, rhyolite, and schist from the EGS Collab site at the SURF in Lead, South Dakota. Fracture toughness was greatest in amphibolite, with schist and rhyolite having similar strengths (${K}_{amphibolite}$ > ${K}_{rhyolite}$ ≈ ${K}_{schist}$). Foliated schist samples followed the fracture toughness sequence: ${K}_{divider}$ > ${K}_{arrester}$  > ${K}_{short transverse}$. Microscopic analysis revealed that in short transverse geometries, fracture propagation was primarily intragranular. In foliated samples, fracture tortuosity was greater in arrester and divider geometries than in short transverse geometries. Fracture toughness was observed to decrease with decreasing loading rate. Because the amphibolite had the greatest fracture toughness of the amphibolite, rhyolite, and schist, we expect greater hydraulic stimulation pressures will be required to initiate fracture propagation in the amphibolite. The schist samples with divider geometries had the greatest fracture toughness of the arrester, divider, and short transverse geometries, so we expect greater hydraulic pressures to initiate fracture propagation in divider geometries. We hypothesize that increased fracture tortuosity and roughness may increase heat exchange in enhanced geothermal systems. However, when fracture tortuosity and roughness increase, fracture permeability may decrease as a result.

## Supplementary Information

Supplementary Information.

## Data Availability

Data used to calculate fracture toughness can be accessed in the supplementary materials.

## List of symbols

$${K}_{IC}$$: Fracture toughness
${P}_{max}$: Maximum diametrical load
*B*: CCNBD sample thickness
$R$: CCNBD sample radius
${Y}_{min}^{*}$: Critical stress intensity factor
$u$, $v$: Dimensionless geometric constants used to calculate ${Y}_{min}^{*}$
α_0_, α_1_, α_B_: Three basic dimensions for the CCNBD geometrical parameters
${a}_{0}$: Half the initial chevron notch length
${a}_{1}$: Half the final chevron notch length
