# Supplementary material for: Fracture toughness of schist, amphibolite, and rhyolite from the Sanford Underground Research Facility (SURF), Lead, South Dakota
Source: Sci Rep. 2022 Sep 24;12:15941. doi: 10.1038/s41598-022-20031-y (PMC9509392; doi:10.1038/s41598-022-20031-y)
Supplement: Supplementary file 1 — Supplementary Information. [file 41598_2022_20031_MOESM1_ESM.docx]

***Supplementary Table S1*** *Arrester, divider, and short transverse geometries as described by the combination of fracture propagation direction and notch direction as being parallel or perpendicular relative to foliation planes.*

|  | | Fracture Propagation Direction Relative to Foliation Planes | |
| --- | --- | --- | --- |
|  |  |  |  |
| Notch Direction Relative to Foliation Planes |  | Short Transverse | - |
|  |  | Divider | Arrester |

***Supplementary Figure S1*** *CCNBD samples before and after fracture toughness tests. (a) Acoustic emission sensors attached to an amphibolite sample which were used to measure diametrical velocities around the sample circumference. (b) Apparatus used to conduct fracture toughness tests. (c) Cracked rhyolite CCNBD after completion of a fracture toughness test*


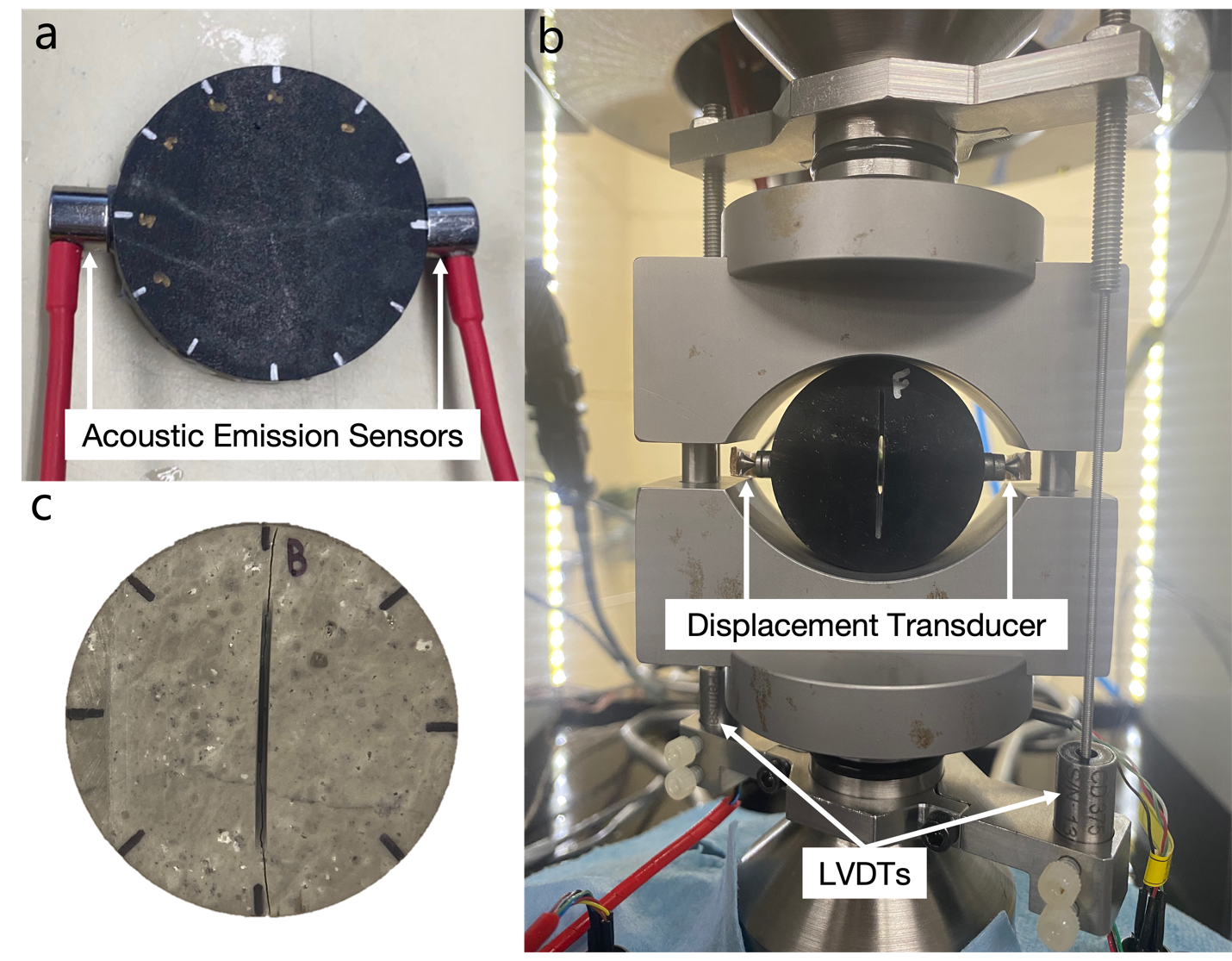


***Supplementary Table S2*** *Sample ID, lithology, notch orientation, loading rate, maximum diametrical load, and fracture toughness for individual tests. Total tests = 50 (A = 15, R = 6, SA = 6, SD = 15, SS = 8). The 15 samples with numerical IDs were previously reported by Ruplinger et al. (2020)^2^.*

| Sample ID | Lithology | R  (mm) | a_0_  (mm) | a_1_  (mm) | Notch Orientation | Loading Rate (μm/s) | Maximum Diametrical Load (kN) | Fracture Toughness (MPa m^1/2^) |
| --- | --- | --- | --- | --- | --- | --- | --- | --- |
| A01 | Amphibolite | 18.92 | 3.85 | 12.62 | Random | 20 | 4.75 | 2.45 |
| A02 | Amphibolite | 18.94 | 5.15 | 13.10 | Random | 20 | 10.22 | 5.45 |
| A03 | Amphibolite | 18.93 | 3.64 | 12.67 | Random | 20 | 7.55 | 3.77 |
| A04 | Amphibolite | 18.93 | 4.95 | 12.94 | Random | 20 | 10.27 | 5.39 |
| A12 | Amphibolite | 18.84 | 4.27 | 12.96 | Random | 20 | 7.00 | 3.54 |
| A13 | Amphibolite | 18.90 | 4.50 | 12.61 | P-wave: Slowest | 20 | 7.61 | 4.02 |
| A14 | Amphibolite | 18.91 | 4.23 | 12.69 | P-wave: Fastest | 20 | 7.55 | 3.82 |
| A15 | Amphibolite | 18.93 | 4.91 | 12.77 | P-wave: Fastest | 20 | 5.43 | 2.88 |
| A17 | Amphibolite | 18.94 | 2.39 | 12.52 | P-wave: Slowest | 20 | 6.90 | 3.42 |
| A18 | Amphibolite | 18.94 | 2.15 | 12.34 | P-wave: Slowest | 20 | 7.47 | 3.62 |
| A19 | Amphibolite | 18.93 | 3.34 | 12.60 | P-wave: Fastest | 20 | 7.10 | 3.50 |
| A20 | Amphibolite | 18.97 | 5.34 | 13.25 | Random | 10 | 4.81 | 2.48 |
| A21 | Amphibolite | 19.03 | 5.68 | 13.36 | Random | 10 | 3.34 | 1.79 |
| A22 | Amphibolite | 18.97 | 5.88 | 13.33 | Random | 10 | 3.33 | 1.81 |
| A23 | Amphibolite | 18.97 | 3.50 | 12.46 | Random | 10 | 4.77 | 2.40 |
| R01 | Rhyolite | 19.01 | 4.52 | 12.52 | Random | 20 | 3.99 | 2.06 |
| R02 | Rhyolite | 19.02 | 2.69 | 12.47 | Random | 20 | 3.59 | 1.81 |
| R03 | Rhyolite | 19.01 | 4.62 | 12.53 | Random | 20 | 4.82 | 2.53 |
| R05 | Rhyolite | 19.05 | 4.15 | 12.40 | Random | 20 | 3.54 | 1.83 |
| R07 | Rhyolite | 18.97 | 4.96 | 12.74 | Random | 10 | 3.82 | 2.02 |
| R08 | Rhyolite | 18.97 | 5.49 | 13.00 | Random | 10 | 3.50 | 1.80 |
| SA01 | Schist | 18.98 | 5.16 | 13.13 | Arrester | 10 | 3.29 | 1.72 |
| SA03 | Schist | 18.98 | 5.23 | 13.02 | Arrester | 10 | 3.65 | 1.94 |
| SA08 | Schist | 18.98 | 2.54 | 12.60 | Arrester | 10 | 3.31 | 1.59 |
| 7.1 | Schist | 19.02 | 7.25 | 13.04 | Arrester | 20 | 4.84 | 2.38 |
| 9.2 | Schist | 18.89 | 7.79 | 12.66 | Arrester | 20 | 4.24 | 2.11 |
| 11.3 | Schist | 18.92 | 6.86 | 12.82 | Arrester | 20 | 3.92 | 1.92 |
| SD01 | Schist | 18.93 | 3.94 | 12.60 | Divider | 10 | 4.48 | 2.25 |
| SD04 | Schist | 18.93 | 5.47 | 13.22 | Divider | 10 | 4.33 | 2.25 |
| SD05 | Schist | 18.93 | 4.92 | 12.96 | Divider | 10 | 4.04 | 2.16 |
| SD06 | Schist | 18.94 | 5.19 | 12.98 | Divider | 10 | 3.97 | 2.12 |
| SD07 | Schist | 18.91 | 5.18 | 13.01 | Divider | 10 | 5.04 | 2.66 |
| SD09 | Schist | 18.92 | 5.22 | 13.27 | Divider | 10 | 4.22 | 2.20 |
| SD10 | Schist | 18.91 | 3.77 | 11.81 | Divider | 10 | 3.59 | 2.05 |
| 1.1 | Schist | 19.06 | 8.14 | 13.06 | Divider | 20 | 5.80 | 2.90 |
| 1.2 | Schist | 19.01 | 6.89 | 12.65 | Divider | 20 | 5.41 | 2.61 |
| 3.1 | Schist | 19.03 | 6.85 | 12.64 | Divider | 20 | 5.93 | 2.89 |
| 3.2 | Schist | 19.03 | 7.64 | 12.72 | Divider | 20 | 5.29 | 2.60 |
| 10.2 | Schist | 19.01 | 7.93 | 13.08 | Divider | 20 | 4.24 | 2.10 |
| 10.3 | Schist | 19.07 | 7.29 | 12.58 | Divider | 20 | 4.83 | 2.49 |
| 12.1 | Schist | 18.94 | 7.30 | 12.18 | Divider | 20 | 6.41 | 3.15 |
| 12.2 | Schist | 18.97 | 7.45 | 12.69 | Divider | 20 | 5.45 | 2.80 |
| SFS08 | Schist | 18.98 | 4.90 | 12.87 | Short Transverse | 10 | 2.78 | 1.44 |
| SFS09 | Schist | 19.08 | 4.51 | 12.58 | Short Transverse | 10 | 3.01 | 1.61 |
| SFS10 | Schist | 18.92 | 4.22 | 12.73 | Short Transverse | 10 | 2.12 | 1.04 |
| SFS11 | Schist | 19.03 | 4.43 | 13.02 | Short Transverse | 10 | 3.38 | 1.68 |
| 2.1 | Schist | 19.04 | 7.39 | 12.75 | Short Transverse | 20 | 3.36 | 1.60 |
| 4.1 | Schist | 19.07 | 6.02 | 12.73 | Short Transverse | 20 | 1.36 | 0.67 |
| 9.1 | Schist | 18.91 | 8.49 | 13.91 | Short Transverse | 20 | 2.50 | 1.33 |
| 11.1 | Schist | 18.94 | 8.33 | 12.80 | Short Transverse | 20 | 1.52 | 0.77 |

***Supplementary Table S3*** *Variation of fracture toughness between sample groups and loading rate.*

| *Sample Group* | *Minimum (MPa m^1/2^)* | *Maximum*  *(MPa m^1/2^)* | *Mean*  *(MPa m^1/2^)* | *Samples Tested* |
| --- | --- | --- | --- | --- |
| *Loading Rate: 20 μm/s* | | | | |
| *Amphibolite* | 2.45 | 5.45 | 3.80 ± 0.91 | 11 |
| *Rhyolite* | 1.81 | 2.53 | 2.06 ± 0.34 | 4 |
| *Schist – Arrester* | 1.92 | 2.38 | 2.14 ± 0.23 | 3 |
| *Schist – Divider* | 2.10 | 3.15 | 2.70 ± 0.32 | 8 |
| *Schist – Short Transverse* | 0.67 | 1.60 | 1.09 ± 0.44 | 4 |
| *Loading Rate: 10 μm/s* | | | | |
| *Amphibolite* | 1.79 | 2.48 | 2.12 ± 0.37 | 4 |
| *Rhyolite* | 1.80 | 2.02 | 1.91 ± 0.15 | 2 |
| *Schist – Arrester* | 1.59 | 1.89 | 1.73 ± 0.15 | 3 |
| *Schist – Divider* | 2.05 | 2.66 | 2.24 ± 0.20 | 7 |
| *Schist – Short Transverse* | 1.04 | 1.68 | 1.44 ± 0.28 | 4 |

***Supplementary Table S4*** *Fracture toughness of Mancos shale in Chandler et al. (2017)^7^ and Chandler et al. (2016)^6^; Marcellus shale in Li, Jin, & Cusatis (2017)^8^; Anvil Point oil shales in Schmidt and Huddle (1977)^5^. “Large” and “Medium” designations for Marcellus Shale indicate the size of samples tested. Oil shales B and D have nominal kerogen contents of 20 and 40 gal/ton, respectively.*

| Sample | Orientation | Fracture Toughness (MPa m^1/2^) |
| --- | --- | --- |
| Mancos Shale (22 °C)  (Chandler et al., 2017) | Divider | 0.56 ± 0.18 |
|  | Arrester | 0.49 ± 0.07 |
|  | Short Transverse | 0.22 ± 0.12 |
| Marcellus Shale – Large  (Li, Jin, & Cusatis, 2017) | Divider | 0.97 ± 0.04 |
|  | Arrester | 0.85 ± 0.06 |
|  | Short Transverse | 0.82 ± 0.04 |
| Marcellus Shale – Medium  (Li et al., 2017) | Divider | 0.85 ± 0.03 |
|  | Arrester | 0.84 ± 0.14 |
|  | Short Transverse | 0.77 ± 0.01 |
| Mancos Shale  (Chandler et al., 2016) | Divider | 0.72 |
|  | Arrester | 0.62 |
|  | Short Transverse | 0.21 – 0.52 |
| Anvil Point Oil Shale Block B  (Schmidt and Huddle, 1977) | Divider | 1.02 – 1.13 |
|  | Arrester | 0.92 – 0.95 |
|  | Short Transverse | 0.75 |
| Anvil Point Oil Shale Block D  (Schmidt and Huddle, 1977) | Divider | 0.64 – 0.67 |
|  | Arrester | 0.60 – 0.61 |
|  | Short Transverse | 0.32 – 0.41 |
